# The Role of CD147 in Pathological Cardiac Hypertrophy Is Regulated by Glycosylation

**DOI:** 10.1155/2022/6603296

**Published:** 2022-01-20

**Authors:** Fang-yuan Zhong, Yi-chao Zhao, Chen-xu Zhao, Zhi-chun Gu, Xi-yuan Lu, Wen-long Jiang, Ling-chen Gao, Wen-li Li, Zi-han Qin, Heng Ge, Jun Pu

**Affiliations:** ^1^Department of Cardiology, Renji Hospital, School of Medicine, Shanghai Jiao Tong University, Shanghai, China; ^2^Department of Pharmacy, Renji Hospital, School of Medicine, Shanghai Jiao Tong University, Shanghai, China

## Abstract

CD147, also known as EMMPRIN or basigin, is a transmembrane glycoprotein receptor that activates matrix metalloproteinases and promotes inflammation. CD147 function is regulated by posttranslational modifications of which glycosylation has attracted the most attention. In this study, we demonstrated that glycosylated CD147 was the dominant form in heart tissue, and its levels were markedly elevated in response to transverse aortic constriction (TAC). Adeno-associated virus 9-mediated, cardiac-specific overexpression of wild-type CD147 in mice significantly promoted pressure overload-induced pathological cardiac remodeling accompanied by augmented oxidative stress and ferroptosis. By contrast, mutations of CD147 glycosylation sites notably weakened these detrimental effects of CD147. Mechanistically, CD147 exacerbated TAC-induced pathological cardiac remodeling via direct binding with the adaptor molecule TRAF2 and subsequent activation of TAK1 signalling, which was dependent on glycosylation of CD147. Collectively, our findings provide the first evidence that CD147 promoted pathological cardiac remodeling and dysfunction in a glycosylation-dependent manner through binding the adaptor protein TRAF2 and activating the downstream TRAF2-TAK1 signalling pathway. Thus, glycosylation of CD147 may be a potent interventional target for heart failure treatment.

## 1. Introduction

Heart failure (HF) is a common final stage of many cardiovascular diseases and is characterised by functional impairment of ventricular filling or ejection [1]. Recent epidemiological studies have shown that HF is currently a substantial public health burden for national medical and health services [2]. Pathological cardiac hypertrophy and adverse ventricular remodeling in response to various extracellular stress stimuli are fundamental morphological changes during the progression of HF [3]. Although great progress has been made in the past decades regarding treatment options for HF, prevalence and hospitalisations are still increasing [4]. Undoubtedly, it is important to identify molecular regulators of pathological cardiac hypertrophy and discover novel therapeutic targets.

Cluster of differentiation (CD) 147 (also known as EMMPRIN and basigin) is encoded by the *BSG* gene and is a versatile transmembrane glycoprotein that promotes matrix metalloproteinase (MMP) activation, myofibroblast differentiation, fibrosis, and oxidative stress [5, 6]. The location of CD147 on the cell surface facilitates binding to multiple inflammatory ligands, such as cyclophilin A (CyPA), CyPB, S100A9, and soluble CD147 (sBSG) itself [7]. Recent reports have demonstrated that CD147 is involved in the development and progression of cardiovascular diseases, including ischemic cardiomyopathy [8, 9], heart failure [10], and atherosclerosis [11]. Of interest, CD147 is posttranslationally modified by N-glycosylation, and the biological function of CD147 is critically regulated by this modification [12]. For example, highly glycosylated but not low-glycosylated CD147 plays a crucial role in MMP induction [13]. Moreover, glycosylation regulates the involvement of CD147 in certain disease states, such as tumour metastasis and cerebral infarction [14–17]. However, the role of CD147 glycosylation in heart disease remains unclear, especially for cardiac remodeling and HF. In this study, we aimed to determine the role of CD147 glycosylation in transverse aortic constriction- (TAC-) induced pathological cardiac remodeling in mice and investigate the underlying mechanisms. Our results demonstrated that CD147 overexpression significantly promoted TAC-induced cardiac remodeling, while glycosylation site mutagenesis notably weakened the detrimental effects of CD147 via suppression of CD147-TRAF2 binding and downstream activation of TAK1 signalling. Thus, glycosylation played a key role in regulating the prohypertrophic effects of CD147, and regulation of CD147 glycosylation may represent a potent interventional target for heart failure treatment.

## 2. Materials and Methods

### 2.1. The Experimental Animals

Male, 8-week-old, wild-type C57BL/6J mice were obtained from Shanghai Model Organisms (Shanghai, China) and housed at 25 ± 2°C with a 12 h light/dark cycle and free access to water and food. All animal experiments were conducted in accordance with the National Institutes of Health Guidelines on the Use of Laboratory Animals and approved by the Institute's Animal Ethics Committee of Shanghai Jiao Tong University.

### 2.2. Adeno-Associated Virus (AAV) Vectors and *In Vivo* Delivery

To examine the role of CD147 glycosylation in pathological cardiac remodeling, AAV-9 vectors expressing mouse wild-type CD147 (OE) or CD147 with 3 mutated glycosylation sites (mutant) were constructed by Hanbio, Inc. (Shanghai, China). Mutagenesis was performed at the three conserved Asn glycosylation sites N44, N154, and N190. An empty vector was used as the control (vehicle). The average viral titres were 3.5 × 10^12^, 3.3 × 10^12^, and 3.1 × 10^12^ viral genomes (vg)/mL for OE, mutant, and vehicle, respectively. Vectors were diluted with sterile saline, and 1 × 10^12^ vg/mL OE or mutant vector in a 50 *μ*L volume was injected into the myocardium in situ using a 30-gauge needle as previously described [18]. Briefly, the animals were anaesthetised using 2% isoflurane, and the hearts were exposed by a left thoracotomy at the 5th intercostal space. The AAV vectors were delivered via direct injection into the left ventricular wall (five sites/heart, 10 *μ*L/site). The same dose of vehicle vector was injected into the hearts of the control mice. The target protein expression in cardiac tissue in a set of mice was analysed 14 days after virus delivery using western blots.

### 2.3. Transverse Aortic Constriction Model

Based on their respective AAV vectors, the mice were assigned to four surgery groups: sham, vehicle, OE, and mutant. Pathological cardiac hypertrophy and remodeling were induced by transverse aortic constriction (TAC) 14 days after AAV injection. TAC was performed as described previously [19, 20]. Briefly, isoflurane-anaesthetised mice were subjected to ligation of the transverse aorta between the innominate and left common carotid arteries with a 27-gauge needle using 6-0 sutures; the identical procedure without constriction served as the sham surgery. Mice with a transverse aortic peak velocity greater than 4000 mm/s assessed by echocardiography were evaluated in our study. The surgeries and subsequent echocardiographic evaluations were performed by individuals blinded to the identity of the mouse genotypes.

### 2.4. TAK1 Inhibitor Treatment *In Vivo*

For TAK1 inhibition, the specific inhibitor 5Z-7-oxozeaenol (5Z-7-ox; #O9890; Sigma; St. Louis, MO, USA) was administered intraperitoneally to wild-type mice overexpressing CD147 (CD147-OE) and mice with mutated CD147 (CD147-Mut) at a dose of 5 mg/kg body weight every three days after TAC surgery. The control group was administered the identical volume of dimethyl sulfoxide (DMSO).

### 2.5. Cardiac Function and Echocardiography

Transthoracic echocardiography was performed to evaluate cardiac function at the indicated times post-TAC surgery using the Vevo 2100 (VisualSonics; Toronto, Canada) imaging system equipped with an MS-550 transducer. Mice were anaesthetised using isoflurane, and heart rates were maintained at approximately 480–520 beats per minute to minimise data deviation during the echocardiographic examination. Dimensional and functional parameters of the left ventricle (LV) were assessed at the level of the papillary muscles using M-mode tracings and averaged using three to five cardiac cycles as previously described [21–23]. Subsequently, an apical four-chamber view was acquired, the peak flow velocities during early diastole (E wave) and end diastole (A wave) were measured, and the E/A, which reflected the left ventricular diastolic function, was calculated.

### 2.6. Cardiovascular Magnetic Resonance (CMR)

CMR was employed to evaluate cardiac function and structure as described recently [24]. All CMR scans were performed with a BioSpec 7T MR system (Bruker BioSpin; Rheinstetten, Germany) equipped with a microimaging accessory and custom-built coils designed specifically for mice. Mice were anaesthetised and maintained with isoflurane via a face mask. The short- and long-axis cine images were acquired using an electrocardiogram-triggered and respiratory-gated gradient echo sequence (TR/TE = 5.2/1.3 ms, flip angle: 15°) with gradient and radiofrequency spoiling. CMR data were analysed off-line with cvi42 software (Circle CVI; Calgary, Alberta, Canada) installed in a CMR image processing workstation.

### 2.7. Histomorphology and Immunohistochemistry

Heart tissue comprising the papillary muscle of the LV was harvested from each mouse and fixed with paraformaldehyde (4%) for 24 h. Then, the hearts were dehydrated, embedded in paraffin, and 5–6 *μ*m thick serial sections were prepared. The slides were stained with haematoxylin-eosin (HE) and wheat germ agglutinin (Invitrogen, Carlsbad, CA, USA) to observe the general morphology of the cardiomyocytes. Picrosirius red (PSR) and Masson's trichrome staining was performed to visualise fibrosis. Image-Pro Plus 6.0 (Media Cybernetics, Inc.; Rockville, MD, USA) was used to measure the myocyte cross-sectional area and percentage of fibrotic tissue in HE- and PSR-stained sections, respectively. For detecting the levels of oxidative stress in the myocardium, immunohistochemistry was performed using anti-nitrotyrosine (sc-32757; Santa Cruz Biotechnology; Dallas, TX, USA) and anti-4-hydroxynonenal (4-HNE; ab46545; Abcam; Cambridge, UK) antibodies.

### 2.8. Western Blot Analysis

Total protein from heart tissue was extracted in accordance with a standard protocol, and a Pierce BCA Protein Assay Kit (Thermo Fisher Scientific; Rockford, IL, USA) was used to determine protein concentrations. Between 30 *μ*g and 60 *μ*g of proteins per lane were separated by SDS-PAGE, transferred to 0.45 *μ*m PVDF membranes, blocked with 5% bovine serum albumin, and incubated with the appropriate primary antibodies overnight at 4°C. Anti-CD147 (ab188190) was purchased from Abcam (Cambridge, UK). Antibodies against GAPDH (2118), phospho(p)-protein kinase B (AKT) (4060), total-AKT (4691), p-p38 AMPK (4511), p38 AMPK (8690), p-JNK (4668), JNK (9258), *α*-SMA (19245), and MMP-2 (40994) were obtained from Cell Signaling Technology (Beverly, MA, USA). Anti-TRAF2 (A0962) and anti-Col3A1 (A3795) were provided by ABclonal (Wuhan, China). Antibodies against GPX4 (sc-166570), COX-2 (sc-19999), ANP (sc-515701), *β*-MHC (sc-53090), p-GSK3*β* (sc-373800), total-GSK3*β* (sc-377213), and COX-2 (sc-19999) were obtained from Santa Cruz Biotechnology. Anti-DDDDK tag antibody (20543-1-AP) was purchased from Proteintech (Wuhan, China). Antibodies against NOX4 (CY5255), NOX1 (AY4775), ACSL4 (CY10198), p-MAP3K7 (Thr187), p-TAK1 (CY6331), and MAP3K7 (TAK1; CY7057) were purchased from Abways Technology, Inc. (Shanghai, China). The protein bands were visualised using enhanced chemiluminescence (Millipore) after incubation with the corresponding secondary antibodies. Deglycosylation assay by PNGaseF (New England Biolab, Beverly, MA) was performed according to the manufacturer's protocol.

### 2.9. Coimmunoprecipitation

After heart tissue proteins were extracted and quantified, a total of 1000 *μ*g protein was subjected to coimmunoprecipitation experiments using Anti-DYKDDDDK IP Resin (L00425; GenScript; Nanjing, China) in accordance with the manufacturer's standard protocol. Briefly, after washing the resin with equilibration buffer, the protein sample was added to the resin, and the mixture was incubated on a shaker overnight at 4°C. The resin-protein complexes were washed and centrifuged the next day. The precipitated proteins were collected, separated using SDS-PAGE, and specific proteins were detected by western blotting using the corresponding antibody.

### 2.10. RNA Isolation and Real-Time PCR

Isolation of total RNA from mouse heart tissue was performed using TRIzol Reagent (Vazyme Biotech, Nanjing, China) in accordance with the manufacturer's protocol. Total RNA (1 *μ*g) was used in a 20 *μ*L reaction to synthesise cDNA using PrimeScript™ RT Master Mix (Perfect Real Time) (Vazyme). Gene expression analyses were performed using TB Green® Premix Ex Taq™ (Tli RNaseH Plus) (Vazyme) and the Light-Cycler 480 Real-Time PCR System (Roche Applied Science; Indianapolis, IN, USA). Expression levels of target genes were expressed as fold change normalised to *Gapdh* levels.

### 2.11. Statistical Analysis

Data were expressed as means ± SEM. Comparisons between the 2 groups were performed using unpaired *t*-tests. Comparisons among multiple groups were performed using one-way or two-way analysis of variance followed by Bonferroni's multiple comparison tests. A two-sided *P* value < 0.05 was considered to be statistically significant.

## 3. Results

### 3.1. CD147 Glycosylation Was Significantly Increased in Response to Pressure Overload

**Figure 1:**
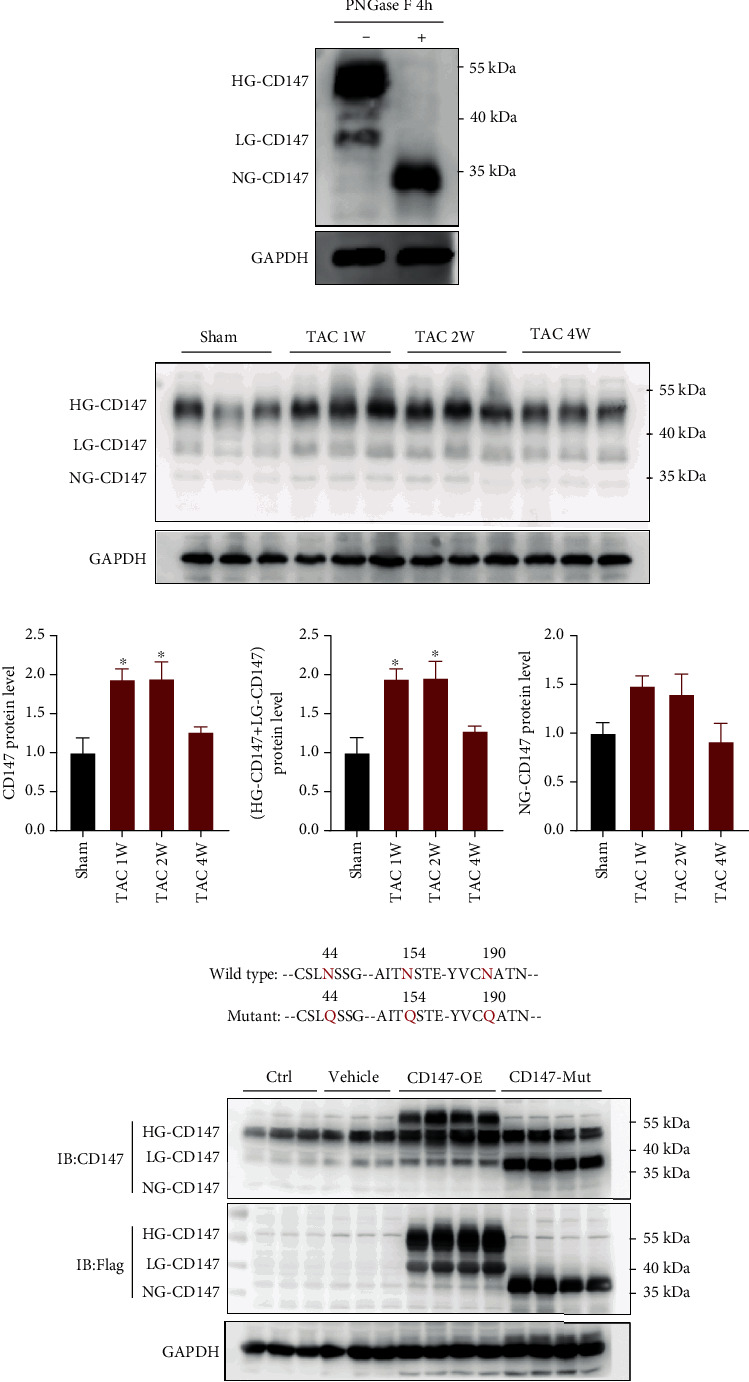
CD147 expression in mouse hearts. Mice overexpressing wild-type CD147 or glycosylation defective CD147 were generated by injecting adeno-associated virus-9 vectors containing the individual constructs into the myocardia. (a) Representative western blot analysis of CD147 expression in mouse hearts treated in the presence or the absence of peptide-N-glycosidase F (PNGase F). Three bands of approximately 32 kDa, 38 kDa, and 50 kDa were detected, which corresponded to nonglycosylated (core protein, NG), low-glycosylated (LG), and high-glycosylated (HG) CD147, respectively. (b) Western blot analysis of CD147 expression in mouse hearts after sham or transverse aortic constriction surgery (*n* = 3/group). (c) Mutagenesis of CD147 glycosylation sites. Three conserved asparagine (Asn) glycosylation sites (N44, N154, and N190) in mouse CD147 were converted to glutamine (Gln) to generate glycosylation-defective CD147 protein. (d) Western blot analysis of overexpression of CD147-WT-3XFlag and CD147-mutant-3XFlag in mouse hearts 14 days after injection with AAV-9 vector alone (vehicle), AAV-9 with wild-type CD147 (CD147-WT), or AAV-9 with glycosylation-defective CD147 (CD147-Mut). It is noteworthy that the bands for glycosylated CD147 (CD147-WT) and mutant nonglycosylated CD147 (CD147-Mut) shifted up in the immunoblot because of the addition of the 3XFlag protein. ^∗^*P* < 0.05 vs. the sham group; ns: not significant.

We first detected the expression of CD147 in the heart by western blot, and three bands of approximately 32 kDa, 38 kDa, and 50 kDa were detected (Figure 1(a)). In order to identify the glycosylated bands of CD147 in the heart, we treated the mouse heart tissue sample with PNGase F, an N-endoglycosidase removing N-linked oligosaccharide chains from glycoprotein, followed by WB. This method is widely used to identify specific glycosylated bands of glycoproteins [25, 26]. Consistent with previous findings, western blot experiment showed that the ~38 kDa and~50 kDa bands of CD147 disappeared and the nonglycosylated CD147 was enhanced at the molecule weight ~32 kDa following PNGase F treatment (Figure 1(a)) [12, 17, 27]. Thus, we proposed that the CD147 bands at ~32 kDa, ~38 kDa, and~50 kDa were corresponding to nonglycosylated (core protein, NG), low-glycosylated (LG), and high-glycosylated (HG) CD147 as reported [12, 17, 27]. We then evaluated whether the expression of CD147 and its glycosylation levels were altered in the pressure overload-induced hypertrophic model. Compared to sham, cardiac CD147 expression was approximately 2-fold higher in heart tissues at 1 week and 2 weeks after TAC and returned to baseline level at 4 weeks after TAC (Figure 1(b)). It is noteworthy that all of these changes mainly occurred in glycosylated CD147 (Figure 1(b)). Collectively, glycosylated CD147 was the dominant expressing form in mouse heart tissue and significantly elevated during hypertrophic process, and this alteration may imply potential function relevance of CD147 glycosylation in cardiac remodeling.

### 3.2. Overexpression of Wild-Type CD147 but Not the Glycosylation Site Mutant Promoted TAC-Induced Cardiac Dysfunction

**Figure 2:**
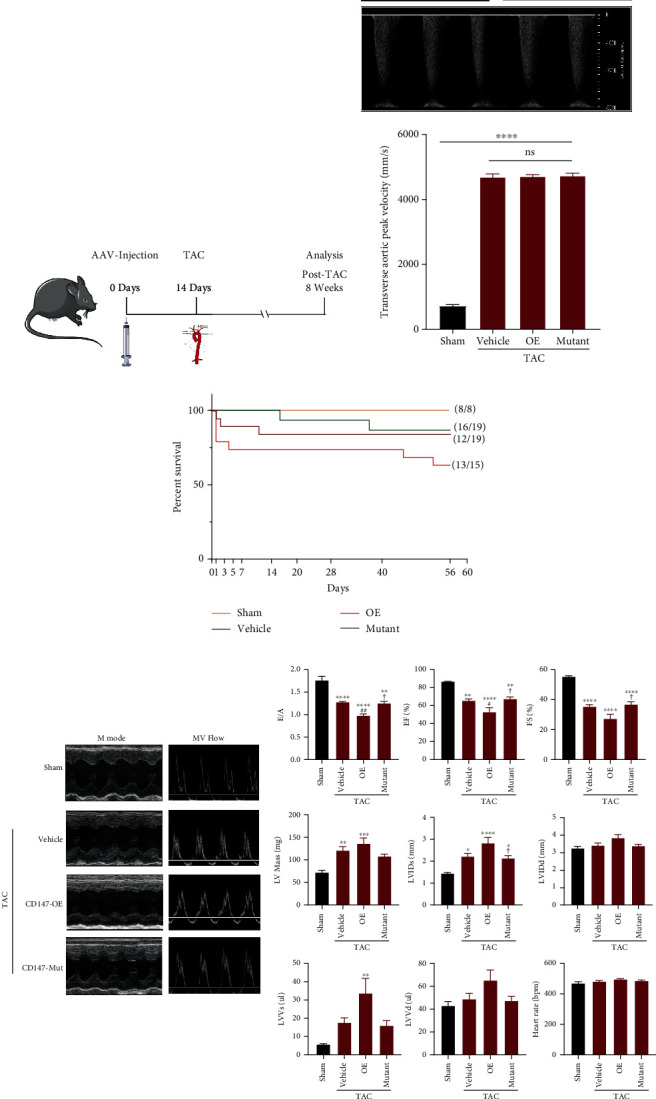
Comparisons of survival rates and cardiac functions among vehicle, CD147-OE, and CD147-mutant mice after transverse aortic constriction (TAC). (a) Schematic illustration of the study. Mice were intramyocardially injected with 1 × 10^12^ viral genomes/mL of AAV-9 vector 14 days before TAC. Hearts from each group were analysed at 8 weeks after TAC. (b) Evaluation of transverse aortic peak velocities by echocardiographic examination (*n* = 5–6 mice/group). Upper panel, representative ultrasonic picture; lower panel, quantitative results. (c) Survival rates 8 weeks after TAC surgery for each group. (d) Echocardiographic analysis of cardiac dysfunction and remodeling. The representative M-mode images of the papillary muscles and maximal mitral valve Doppler flow profiles in the apical 4-chamber view are shown in the left panel. Statistical analysis of the echocardiographic parameters is shown in the right panel (*n* = 8–9 mice/group). ^∗^*P* < 0.05 vs. the sham group; ^∗∗^*P* < 0.01 vs. the sham group; ^∗∗∗^*P* < 0.001 vs. the sham group; ^∗∗∗∗^*P* < 0.0001 vs. the sham group; ^#^*P* < 0.05 vs. the vehicle group; ^##^*P* < 0.01 vs. the vehicle group; ^✟^*P* < 0.05 vs. the OE group; ^✟✟^*P* < 0.01 vs. the OE group. The data are expressed as the means ± SEM. E/A: transmitral filling peak velocity/atrial wave velocity; EF: ejection fraction; FS: fractional shortening; LV mass: left ventricular mass; LVIDs: left ventricular internal diameter during the systolic phase; LVIDd: left ventricular internal diameter during the diastolic phase; LVVs: left ventricular end-systolic volume; LVVd: left ventricular end-diastolic volume.

To investigate the role of wild-type CD147 and its glycosylation site mutant in TAC-induced cardiac hypertrophy, we constructed AAV-9 vectors overexpressing WT CD147 or its glycosylation mutant (Figure 1(c)) [27]. We delivered these vectors by intramyocardial injections (Supplementary Figure S1) [18], which resulted in successful overexpression of either WT or mutant CD147 (Figure 1(d)). Following TAC surgery, pulse Doppler demonstrated that the transverse aortic peak velocity was comparable among the vehicle, CD147-OE, and CD147-mutant groups of mice (Figure 2(b)). However, 8 weeks after TAC surgery, overexpression of WT CD147 resulted in a survival rate of 63.16% compared with 84.21% in the vehicle group. By contrast, overexpression of the mutant CD147 increased the survival rate to 86.67% compared with that of the CD147-OE group (Figure 2(c)). The CD147-OE mice demonstrated significantly worse cardiac diastolic and systolic functions, as indicated by the decreases in the E wave/A wave (E/A), ejection fraction, and fractional shortening compared with time-matched mice in the vehicle group. These detrimental effects were reversed in mice overexpressing CD147 with glycosylation site mutations. Moreover, CD147-OE mice exhibited augmented LV enlargement (increases in LV internal diameter at diastolic and systolic phases, LV volume at diastolic and systolic phases), and increased LV mass compared with the Vehicle control group. By contrast, CD147-mutant mice showed improvements in LV enlargement after TAC compared with the CD147-OE group (Figure 2(d)).

### 3.3. Overexpression of WT CD147 but Not Its Glycosylation Site Mutant Exacerbated Pathological Cardiac Hypertrophy

**Figure 3:**
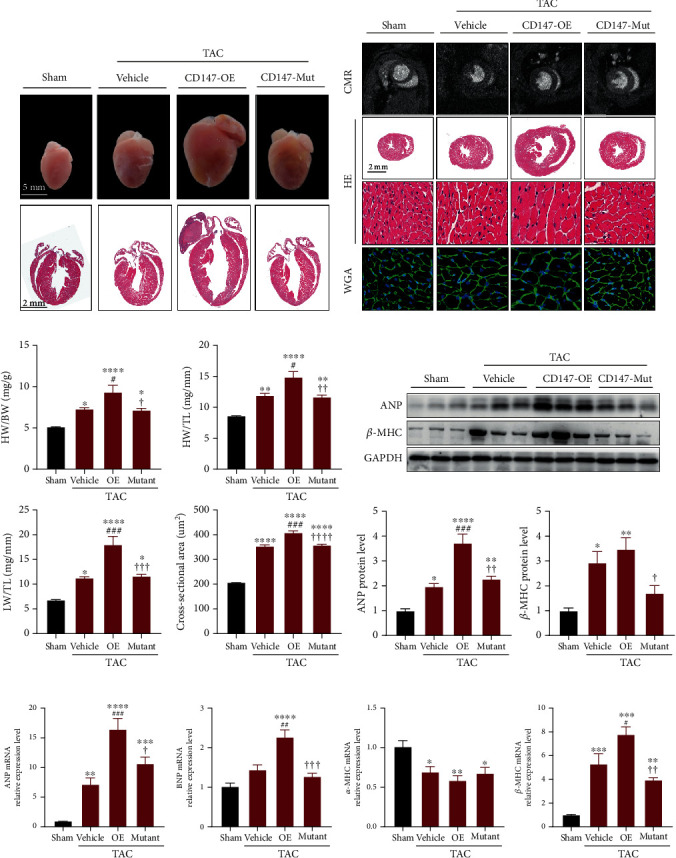
Glycosylation site mutations mitigated the prohypertrophic effects of CD147. (a) Representative images of the hearts and HE staining of four-chamber view sections. (b) Representative images of CMR examinations, HE staining, and WGA staining of heart sections from the indicated groups. (c) Statistical results for HW/BW, HW/TL, LW/TL, and myocyte cross-sectional areas for the indicated groups (*n* = 6–8 mice/group). (d) Western blot analysis of ANP and *β*-MHC in murine hearts from the indicated groups 8 weeks after transverse aortic constriction (TAC) surgery (*n* = 6 mice/group). Upper panel, representative blots. Lower panel, quantitative results. (e) mRNA levels of *Anp*, *Bnp*, *α-Mhc*, and *β-Mhc* in the indicated groups (*n* = 6 mice/group). Results were normalised to *Gapdh* levels and converted to fold induction relative to the sham group. ^∗^*P* < 0.05 vs. the sham group; ^∗∗^*P* < 0.01 vs. the sham group; ^∗∗∗^*P* < 0.001 vs. the sham group; ^∗∗∗∗^*P* < 0.0001 vs. the sham group; ^#^*P* < 0.05 vs. the vehicle group; ^##^*P* < 0.01 vs. the vehicle group; ^###^*P* < 0.001 vs. the vehicle group; ^✟^*P* < 0.05 vs. the OE group; ^✟✟^*P* < 0.01 vs. the OE group; ^✟✟✟^*P* < 0.001 vs. the OE group. CMR: cardiovascular magnetic resonance; HE: haematoxylin-eosin; WGA: wheat germ agglutinin; HW: heart weight; BW: body weight; LW: lung weight; TL: tibia length; ANP: atrial natriuretic peptide; BNP: brain natriuretic peptide; *β*-MHC: myosin heavy chain *β*; *α*-MHC: myosin heavy chain *α*.

To determine the effects of CD147 and its glycosylation on pathological cardiac hypertrophy, we harvested heart tissues for further studies after echocardiographic and CMR examination. As shown in Figures 3(a) and 3(b), macropathological examination,HE staining and WGA staining of cardiac cross-sections demonstrated that CD147-OE significantly promoted TAC-induced cardiac dilation and increasement of cross-sectional areas of cardiomyocytes, and the CD147-mutant markedly blunted these changes. Furthermore, TAC caused marked pathological cardiac hypertrophy as indicated by the increased ratios of heart weight to body weight, heart weight to tibia length, and lung weight to tibia length compared with those in the sham group. Compared with the vehicle group, overexpression of WT CD147 significantly increased these parameters, suggesting exacerbation of cardiac hypertrophy; in contrast, the CD147 glycosylation mutant significantly improved these measurements compared with CD147-OE mice (Figure 3(c)). This enlargement was accompanied by significantly increased protein and mRNA expression of hypertrophic markers (atrial natriuretic factor, brain natriuretic peptide, and *β*-MHC) in the CD147-OE group compared with that in the vehicle group, which were reversed in the CD147-mutant group (Figures 3(d) and 3(e)). Taken together, CD147 overexpression significantly worsened pathological cardiac hypertrophy compared with the vehicle group, and the glycosylation-defective CD147 abrogated these detrimental effects when compared with wild-type CD147 overexpression.

### 3.4. Overexpression of WT CD147 but Not Its Glycosylation Site Mutant Increased TAC-Induced Cardiac Fibrosis

**Figure 4:**
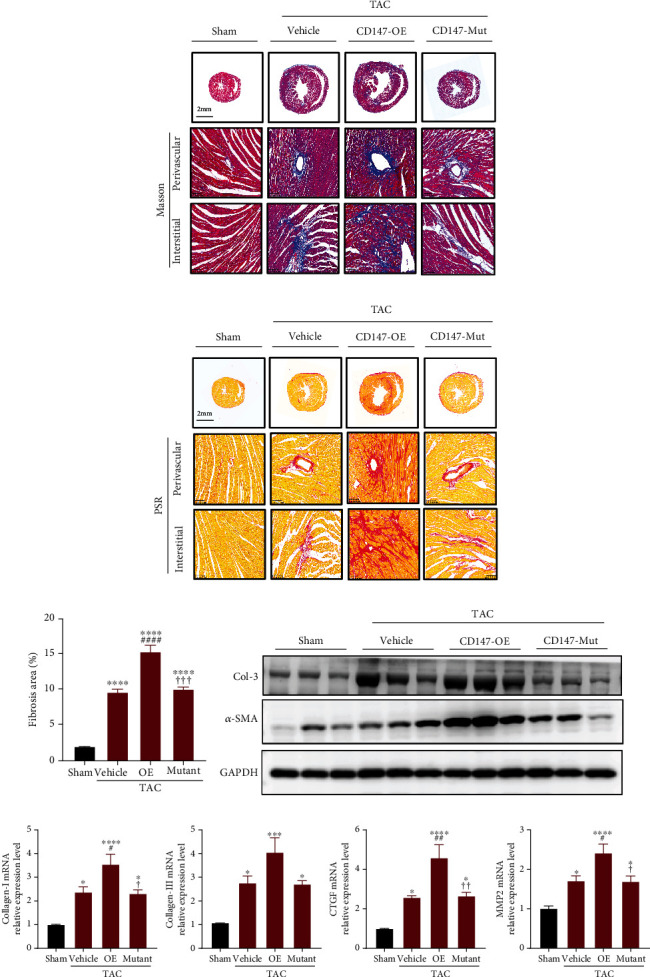
Defective CD147 glycosylation improved profibrotic effects of CD147 in mouse hearts after transverse aortic constriction (TAC). (a) Representative images of heart sections from the different groups stained with Masson's trichrome. (b) Representative images of heart sections stained with PSR. (c) Quantification of the percentage of the left ventricular fibrotic area (*n* = 6 mice/group with 4–6 visual fields per mouse). (d) Representative western blot analysis of collagen-3 (COL-3) and *α*-SMA in cardiac tissue from the indicated groups (*n* = 3). (e) Real-time polymerase chain reaction analysis of collagen-1, collagen-3, *Ctgf*, and *Mmp2* mRNA expression levels in the indicated groups (*n* = 5–6). ^∗^*P* < 0.05 vs. the sham group; ^∗∗^*P* < 0.01 vs. the sham group; ^∗∗∗^*P* < 0.001 vs. the sham group; ^∗∗∗∗^*P* < 0.0001 vs. the sham group; ^#^*P* < 0.05 vs. the vehicle group; ^##^*P* < 0.01 vs. the vehicle group; ^###^*P* < 0.001 vs. the vehicle group; ^####^*P* < 0.0001 vs. the vehicle group; ^✟^*P* < 0.05 vs. the OE group; ^✟✟^*P* < 0.01 vs. the OE group, ^✟✟✟^*P* < 0.001 vs. the OE group. PSR: picrosirius red; COL-3: collagen-3; *α*-SMA: *α*-smooth muscle actin; CTGF: connective tissue growth factor; MMP2: matrix metalloproteinase 2.

The fibrotic response is the main cause of systolic and diastolic dysfunction. We investigated the role of CD147 glycosylation in TAC-induced cardiac fibrosis. Heart sections stained with PSR or Masson's trichrome showed markedly increased perivascular and interstitial fibrosis in the CD147-OE mice after TAC compared with fibrosis in the vehicle group. In contrast, the CD147-mutant group showed overt improvement of pathological fibrosis compared with that in the CD147-OE group (Figures 4(a)–4(c)). Consistently, increased collagen deposition in the heart tissue from CD147-OE mice was accompanied by upregulated protein and mRNA expression of the fibrosis markers collagen I, collagen III, connective tissue growth factor, *α*-smooth muscle actin, and MMP-2, which were distinctly downregulated in CD147-mutant mice when compared with CD147-OE mice (Figures 4(d) and 4(e)).

### 3.5. Overexpression of WT CD147 but Not Its Glycosylation Mutant Aggravated TAC-Induced Myocardial Oxidative Stress and Ferroptosis

**Figure 5:**
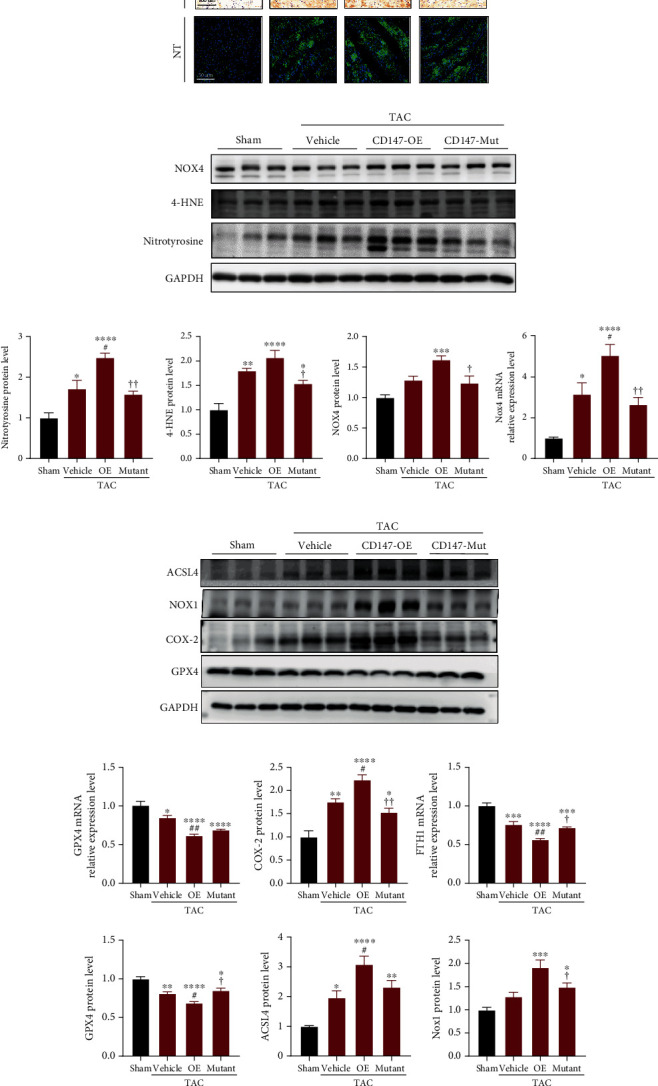
CD147 augmented pressure overload-induced oxidative stress and ferroptosis in a glycosylation-dependent manner. (a) Representative immunostaining for 4-hydroxynonenal (4-HNE) and nitrotyrosine (NT) in mouse myocardia from the indicated groups. (b) Representative immunoblotting and (c) statistical analysis for NT, 4-HNE, and NOX4 protein expression. Results were normalised to GAPDH levels (*n* = 5–6 mice/group). (d) Representative western blotting of ferroptosis-related proteins, including ACSL4, NOX1, COX-2, and GPX4; GAPDH served as the loading control (*n* = 5–6 mice/group). (e) Quantitative analysis of ferroptosis-related gene expression by real-time polymerase chain reaction or protein expression by western blot in the different groups. ^∗^*P* < 0.05 vs. the sham group; ^∗∗^*P* < 0.01 vs. the sham group; ^∗∗∗^*P* < 0.001 vs. the sham group; ^∗∗∗∗^*P* < 0.0001 vs. the sham group; ^#^*P* < 0.05 vs. the vehicle group; ^##^*P* < 0.01 vs. the vehicle group; ^✟^*P* < 0.05 vs. the OE group; ^✟✟^*P* < 0.01 vs. the OE group. 4-HNE: 4-hydroxynonenal; NT: nitrotyrosine; NOX4: NADPH oxidase 4; ACSL4: acyl-CoA synthetase long-chain family member 4; NOX1: NADPH oxidase 1; COX-2: cyclooxygenase-2; GPX4: glutathione peroxidase 4.

Oxidative stress is a crucial trigger of pathological cardiac hypertrophy and heart failure [28]. Pathological hypertrophic stimuli strikingly triggered the production of peroxide by-products of proteins (nitrotyrosine) and lipids (4-HNE), which was demonstrated by immunostaining and immunoblotting (Figures 5(a) and 5(b)). As expected, CD147-OE exacerbated the production of reactive oxygen species (ROS) in heart tissue in response to pressure overload, which was significantly inhibited by mutating the CD147 glycosylation sites. NADPH oxidases (NOXs) are the major enzymes responsible for the production of superoxide (O_2_^−^) or hydrogen peroxide (H_2_O_2_), which are the main sources of ROS in the heart [29]. Among them, NOX4 plays a critical role in TAC-induced pathological cardiac remodeling [30]. Pressure overload for 8 weeks significantly increased the protein and mRNA expression levels of NOX4 in CD147-OE mice, which was markedly reversed by glycosylation site mutations (Figures 5(b) and 5(c)).

Based on the above-mentioned roles for CD147 in oxidative stress and lipid peroxidation, we further explored its role in ferroptosis, a type of programmed cell death dependent on the accumulation of lipid peroxide and iron [31]. Cyclooxygenase-2 (COX-2), also known as prostaglandin-endoperoxide synthase 2, is a well-accepted marker of ferroptosis [32, 33]. In accordance with previous reports [34, 35], the protein expression of COX-2 markedly increased in TAC-induced pathological cardiac hypertrophy and was further upregulated in the hypertrophic hearts of CD147-OE mice (Figure 5(d)). Intriguingly, the CD147 glycosylation mutant mitigated the upregulation of COX-2 in response to TAC. Furthermore, sustained pressure overload reprogrammed other ferroptosis-related genes in mouse hearts. In line with expectations, compared with the vehicle group, overexpression of CD147 significantly increased the protein or mRNA expression levels of proferroptotic genes (ACSL4 and NOX1) and decreased the expression of ferroptosis inhibitors (GPX4 and FTH1). These results were significantly reversed by overexpression of the CD147 glycosylation mutant compared with that of the CD147-OE mice (Figures 5(d) and 5(e)).

### 3.6. Glycosylation Regulated CD147-TRAF2 Binding and Subsequent Activation of TRAF2-TAK1 Signalling

**Figure 6:**
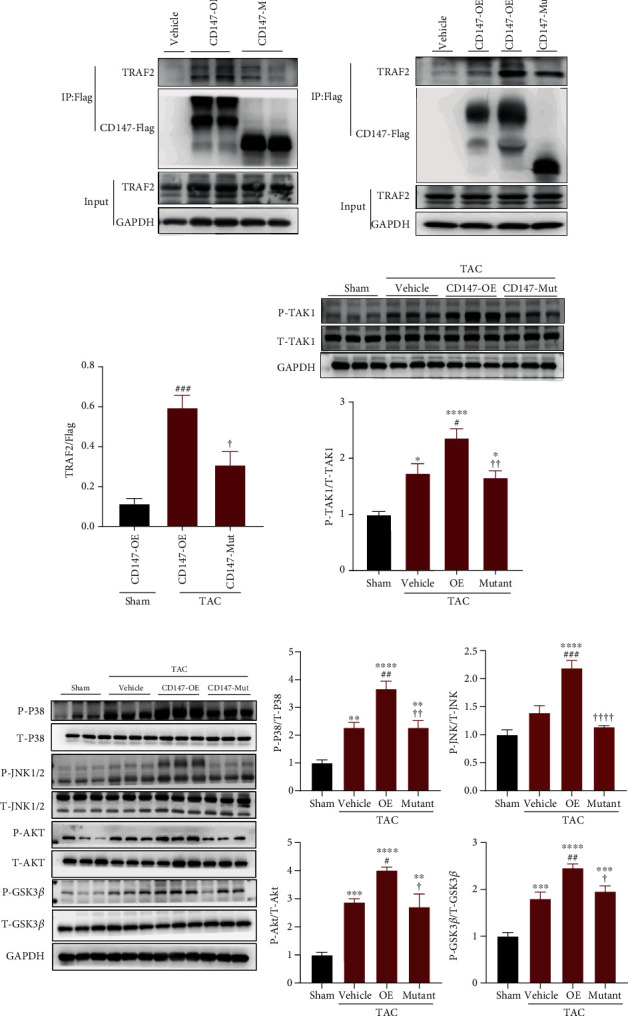
CD147 glycosylation regulated CD147-TRAF2 binding and subsequent activation of TRAF2-TAK1 signalling. (a) TRAF2 binding with CD147-OE and CD147-mutant was observed under physical conditions (*n* = 2). Flag-tagged CD147-OE or CD147-mutant were expressed in mouse hearts. Proteins interacting with CD147 in tissue lysate were immunoprecipitated with anti-DYKDDDDK IP Resin and analysed by western blot using an anti-TRAF2 antibody. (b) Representative image of immunoprecipitation and (c) quantitative analysis of the recruitment of TRAF2 to CD147-OE and CD147-mutant proteins in response to TAC (*n* = 4–5). (d) Representative western blots and quantitative results of phosphorylated and total TAK1 protein levels in hearts from the indicated mouse groups after TAC (*n* = 5–6). (e) Representative western blots and quantitative results for phosphorylated and total protein levels of p38, AMPK, AKT, and glycogen synthase kinase 3*β* (GSK3*β*) in hearts from the different mouse groups after TAC (*n* = 5–6). ^∗^*P* < 0.05 vs. the sham group; ^∗∗^*P* < 0.01 vs. the sham group; ^∗∗∗^*P* < 0.001 vs. the sham group; ^∗∗∗∗^*P* < 0.0001 vs. the sham group; ^#^*P* < 0.05 vs. the vehicle group; ^##^*P* < 0.01 vs. the vehicle group; ^✟^*P* < 0.05 vs. the OE group; ^✟^*P* < 0.01 vs. the OE group.

Signal transduction begins with cell-surface receptors and is controlled by adaptor proteins, which facilitate the creation of signalling complexes by regulating protein-protein interactions [36]. CD147 is a well-known transmembrane receptor that functions via binding with downstream molecules [5]; therefore, we investigated the intracellular CD147 signalling pathways. A motif analysis of CD147 (http://elm.eu.org/) showed a potential TRAF2-binding site in its cytoplasmic domain (Supplementary Figure S2(a)). Furthermore, a recent study demonstrated that the adaptor protein TRAF2 enhanced cardiac hypertrophy and left ventricular dysfunction in mice in response to TAC [37]. To clarify the relationship between CD147 and TRAF2 in the current model, AAV9-mediated Flag-tagged wild-type CD147 and glycosylation defective CD147 were expressed in cardiomyocytes of mice. Immunoprecipitation analysis indicated that overexpressed WT CD147 bound with TRAF2, while CD147-TRAF2 binding was significantly attenuated by the glycosylation site mutations (Figure 6(a)). We then evaluated the expression levels of cardiac TRAF2 in response to pressure overload. Interestingly, TRAF2 expression was significantly increased at weeks 1 and 2 after TAC and declined nearly to baseline by week 4, which positively correlated with CD147 expression in heart tissues after TAC (Supplementary Figure S2 (b-d)). Subsequently, we confirmed that CD147-TRAF2 binding was significantly increased in response to TAC and was markedly reduced by mutations of the CD147 glycosylation sites (Figures 6(b) and 6(c)).

To further investigate the role of CD147 glycosylation in TRAF2-mediated signalling, we determined whether CD147 glycosylation regulated transforming growth factor beta-activated kinase 1 (TAK1), a direct downstream target of TRAF2 [38]. Our results clearly revealed that the phosphorylated levels of TAK1 were significantly increased in CD147-OE mice compared with that in the vehicle group after TAC surgery. By contrast, glycosylation site mutations reversed the effects of CD147-OE on TAK1 phosphorylation (Figure 6(d)). These results indicated that TRAF2-TAK1 signalling is regulated by CD147 glycosylation. The p38/JNK1/2 [39] and AKT/GSK3*β* [40–42] signalling pathways are downstream targets of TAK1 and are implicated in cardiac hypertrophy and heart failure [39, 43]. Therefore, we investigated whether these signalling pathways were also regulated by CD147 glycosylation. Our results demonstrated that the expression levels of phosphorylated p38, JNK1/2, AKT, and GSK3*β* were significantly higher in CD147-OE mice than those in the vehicle group after TAC surgery, and glycosylation site mutations reversed the effects of CD147 overexpression on these signalling kinases (Figure 6(e)). Subsequently, we treated CD147-OE mice with a specific pharmacological TAK1 inhibitor (5Z-7-ox, 5Z) after TAC surgery and this treatment significantly blocked TAK1 activity as demonstrated by the reduced level of phosphorylated TAK1 (Supplementary Figure S3). Furthermore, blocking TAK1 activity abated the activation of p38, JNK1/2, and AKT signalling in CD147-OE mice after TAC surgery when compared with the DMSO-treated controls (Supplementary Figure S3). These results indicated that TRAF2-TAK1 signalling may be involved in exacerbated effects of glycosylated CD147 in cardiac hypertrophy.

### 3.7. TRAF2-TAK1 Signalling Mediated the Prohypertrophic Response of Glycosylated CD147 *In Vivo*

**Figure 7:**
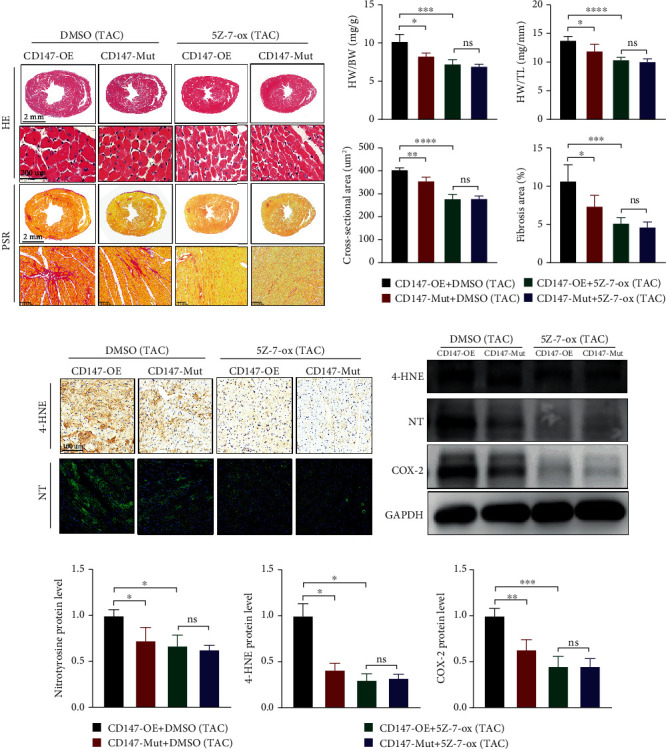
Glycosylated CD147 promoted pathological cardiac hypertrophy that was dependent on TRAF2-TAK1 signalling. (a) Heart sections were stained with HE or PSR to measure cardiomyocyte size or fibrotic area in the indicated groups, respectively. (b) Statistical results for HW/BW, HW/TL, myocyte cross-sectional areas, and fibrotic areas in the indicated groups (*n* = 6–8 per group). (c) Representative immunostaining for myocardial 4-hydroxynonenal (4-HNE) and nitrotyrosine (NT) in the indicated groups. (d) Representative immunoblotting and (e) statistical analysis of NT, 4-HNE, and COX-2. Results were normalised to GAPDH levels (*n* = 3–4). ^∗^*P* < 0.05; ^∗∗^*P* < 0.01; ^∗∗∗^*P* < 0.001; ^∗∗∗∗^*P* < 0.0001; ns: not significant. HE: haematoxylin-eosin; PSR: picrosirius red; HW: heart weight; BW: body weight; TL: tibia length.

To determine whether TRAF2-TAK1 signalling was required for the prohypertrophic effects of glycosylated CD147 post-TAC, we treated CD147-OE mice and CD147-mutant mice with the TAK1 inhibitor 5Z-7-ox or DMSO as a control. As shown in Figures 7(a) and 7(b), 5Z-7-ox treatment reduced the hypertrophic response caused by CD147 glycosylation, and comparable heart weights, cardiomyocyte sizes, and fibrotic areas were observed between TAK1 inhibitor-treated CD147-OE and CD147-mutant mice. Moreover, we investigated whether TRAF2-TAK1 signalling was involved in the prooxidative and proferroptosis actions of CD147. As expected, the prooxidative and proferroptosis role of glycosylated CD147 in pathological cardiac hypertrophy was significantly blunted in mice treated with the TAK1 inhibitor (Figures 7(c) and 7(e)). Taken together, the prohypertrophic effects of glycosylated CD147 are likely regulated by TRAF2-TAK1 signalling.

## 4. Discussion

The present study provided the following new insights into the regulatory role of CD147 glycosylation in pathological cardiac hypertrophy: (a) cardiac CD147 overexpression promoted cardiac maladaptive hypertrophy and remodeling and increased oxidative stress and ferroptosis in response to pressure overload, all of which were reversed by mutations in CD147 glycosylation sites; (b) mechanistic investigations using the TAC-induced HF model revealed that CD147 glycosylation promoted cardiac maladaptive hypertrophy via enhancement of CD147 interactions with the adaptor protein TRAF2 and activation of TRAF2-TAK1 signalling. Collectively, the current study demonstrated for the first time that the role of CD147 in pathological cardiac hypertrophy is regulated by glycosylation, and CD147 glycosylation may represent a novel interventional target for treating pathological cardiac remodeling.

CD147 is a member of the immunoglobulin superfamily and was initially described as a stimulator of MMP-1 production in fibroblasts [44]. CD147 plays well-characterised roles in tumour metastasis, angiogenesis, and chemoresistance [45]. In addition, numerous studies have documented the significance of CD147 in various physiological processes, such as spermatogenesis, fertilisation, neural networks, and retinal development, and in several pathological conditions, including rheumatoid arthritis and infections by malarial parasites and viruses [12]. CD147 has attracted attention for its proposed role in the development and progression of cardiovascular diseases [5]. As reported, CD147 and its ligand CyPA are mediators of inflammatory and oxidative stress after myocardial ischemia and reperfusion [8]. In a mouse model of hypoxia-induced pulmonary hypertension, CD147 promoted inflammation and vascular smooth muscle cell proliferation [46]. Moreover, functional blockage of CD147 ameliorated atherosclerosis in ApoE (-/-) mice by downregulating MMP activity [47]. Notably, a significant biological property of CD147 is its high level of glycosylation. The N-glycosylation modification not only contributes to approximately half the size of the mature molecule but also regulates CD147-mediated biological functions [12]. Glycosylation was shown to be critical for CD147-regulated MMP production and activation in brain injury and tumour metastasis [14, 16]. In the present study, we demonstrated that CD147 promoted pathological cardiac remodeling and dysfunction in a glycosylation-dependent manner. Our study provides novel insights into the pathophysiological significance of CD147 and its glycosylation status in cardiovascular disease.

In response to TAC surgery, the heart initially undergoes a compensatory response to pressure overload characterised by an increase in cardiomyocyte size and thickening of the ventricular walls in mice [48]. However, sustained hemodynamic stress will result in ventricular dilation, failure of relaxation and contraction ability, and eventually leading to HF [3]. During the pathogenesis of cardiac hypertrophy and transition to HF, the overproduction of ROS resulting in oxidative stress has been well recognised as a crucial trigger [28, 49, 50]. Excessive ROS causes DNA damage, protein peroxidation, and cell death, which have been demonstrated in cardiovascular disease [51]. Specifically, ROS can directly impair contractile function by oxidising proteins that are central to excitation-contraction coupling [52]. These detrimental effects can enhance the progression of cardiac hypertrophy and heart failure [52]. It is worth noting that excessive ROS can induce lethal lipid peroxidation, leading to membrane destabilisation and ferroptotic cell death [53, 54]. Ferroptosis is a recently identified form of iron-dependent cell death that is distinct from apoptosis and necroptosis [53]. Importantly, ferroptosis has been implicated in the pathological process associated with ROS-induced heart tissue injury [32, 35, 55]. Previous studies have demonstrated that ferroptosis can take place in adult cardiomyocytes and is implicated in the pathogenesis of doxorubicin- and ischemia/reperfusion- (I/R-) induced cardiomyopathy [32]. Moreover, ferroptosis was reported to be an important process involved in cardiomyocyte loss during sustained pressure overload [34, 35]. For example, Ito et al. demonstrated that an inhibitor of ferroptosis, ferrostatin-1, significantly mitigated the development of pressure overload-induced dilated cardiomyopathy in wild-type mice [35]. In our study, we demonstrated that CD147 increased oxidative stress and ferroptosis in response to pressure overload, which were reversed by CD147 glycosylation site mutations. Additionally, a previous report showed that CD147 promoted myocardial ischemia and reperfusion injury by enhancing oxidative stress [8]. Thus, we conclude that pathological cardiac remodeling exacerbated by glycosylated CD147 may be attributed to enhanced oxidative stress and ferroptotic death.

As a transmembrane glycoprotein, CD147 primarily functions by binding with different molecular partners [7]. For example, through its association with certain monocarboxylate transporters, CD147 was shown to act as a key metabolic regulator [56]. As a cell surface signalling receptor, CD147 receives stimulation from inflammatory factors, including CyPA, CyPB, and S100A9 [5]. In response to inflammatory stimuli, upregulation of CD147 mediates leukocyte infiltration by binding to E-selectin [57]. In our study, we found that CD147 can directly bind with TRAF2, and this binding was enhanced in hypertrophic hearts. Notably, as a key component of the tumour necrosis factor receptor 1 signalling complex, TRAF2 has received considerable attention because of its pivotal role in cardiac pathophysiology [37, 58, 59]. It has been shown that transgenic mice expressing high levels of TRAF2 developed adverse cardiac remodeling and heart failure [59]. TRAF2 expression was increased in failing mouse hearts, and both TRAF2 and its direct downstream signalling partner TAK1 accelerated pressure overload-induced cardiomyopathy and oxidative stress-mediated injury [37, 39, 60]. We demonstrated that CD147 not only interacted with TRAF2 but also activated downstream signalling, as indicated by marked upregulation of phosphorylated TAK1, p38, JNK1/2, and AKT in CD147-OE mice. Interestingly, the CD147-TRAF2 interaction and subsequent TRAF2-TAK1 signalling activation were pivotally regulated by CD147 glycosylation, since defective glycosylation of CD147 significantly abolished this interaction and activation of downstream signalling in hypertrophic hearts. Similarly, the regulatory effects of N-glycosylation on the interaction of CD147 with other proteins are well recognised [12]. Glycosylation regulates the association of CD147 with E-selectin during leukocyte infiltration during an inflammatory response and with integrin *β*1 in hepatocellular carcinoma metastasis [16, 57]. By using the specific TAK1 inhibitor 5Z-7-ox, we confirmed that glycosylated CD147 promoted pathological cardiac hypertrophy through TRAF2-TAK1 signalling. Taken together, CD147 glycosylation mediated the binding of TRAF2 to CD147, subsequently activated downstream TAK1 signalling, and enhanced oxidative stress and ferroptosis, thereby promoting the progression of maladaptive cardiac hypertrophy.

The current findings, along with previous studies indicating important roles of CD147 in atherosclerosis [47], pulmonary artery hypertension [46], ischemic cardiomyopathy [8], and heart failure [10], suggest a key role for CD147 in cardiovascular pathophysiology and as an important therapeutic target. However, because CD147 plays pleiotropic molecular roles in various physiological conditions, directly targeting CD147 may cause severe side effects and tissue injury, as demonstrated by the low survival rate and dysfunction of multiple organs in mice lacking CD147 [61, 62]. Therefore, the current findings demonstrated the regulatory role of CD147 glycosylation in cardiac function and suggest that manipulating CD147 glycosylation may represent an alternative potential strategy to attenuate the development of adverse cardiac remodeling and dysfunction.

## 5. Conclusions

The present study revealed a previously unrecognised role for CD147 glycosylation in pressure overload-induced cardiac maladaptive remodeling via the regulation of TRAF2-TAK1 signalling. We revealed a novel, direct connection between CD147 and TRAF2-TAK1 signalling that is CD147 glycosylation-dependent and provide new insights into the molecular basis of cardiac hypertrophy and heart failure. Our data suggest that regulation of CD147 glycosylation may be a potential interventional strategy for heart failure treatment.

## Data Availability

The data used to support the findings of this study are available from the corresponding author upon request.

## Supplementary Materials

Supplementary Materials Supplementary Figure S1: schema for intramyocardial injection of adeno-associated virus in mouse hearts. Supplementary Figure S2: TRAF2 was upregulated in mouse hearts after sustained pressure overload. Supplementary Figure S3: blocking TAK1 activity reduced the downstream activation of CD147-mediated TRAF2-TAK1 signalling. Supplementary Table S1: mouse primer sequences for qRT-PCR.
