# Supplementary material for: The Role of CD147 in Pathological Cardiac Hypertrophy Is Regulated by Glycosylation
Source: Oxid Med Cell Longev. 2022 Jan 20;2022:6603296. doi: 10.1155/2022/6603296 (PMC8794662; doi:10.1155/2022/6603296)
Supplement: Supplementary Materials — Supplementary Figure S1: schema for intramyocardial injection of adeno-associated virus in mouse hearts. Supplementary Figure S2: TRAF2 was upregulated in mouse hearts after sustained pressure overload. Supplementary Figure S3: blocking TAK1 activity reduced the downstream activation of CD147-mediated TRAF2-TAK1 signalling. Supplementary Table S1: mouse primer sequences for qRT-PCR. [file 6603296.f1.pdf]

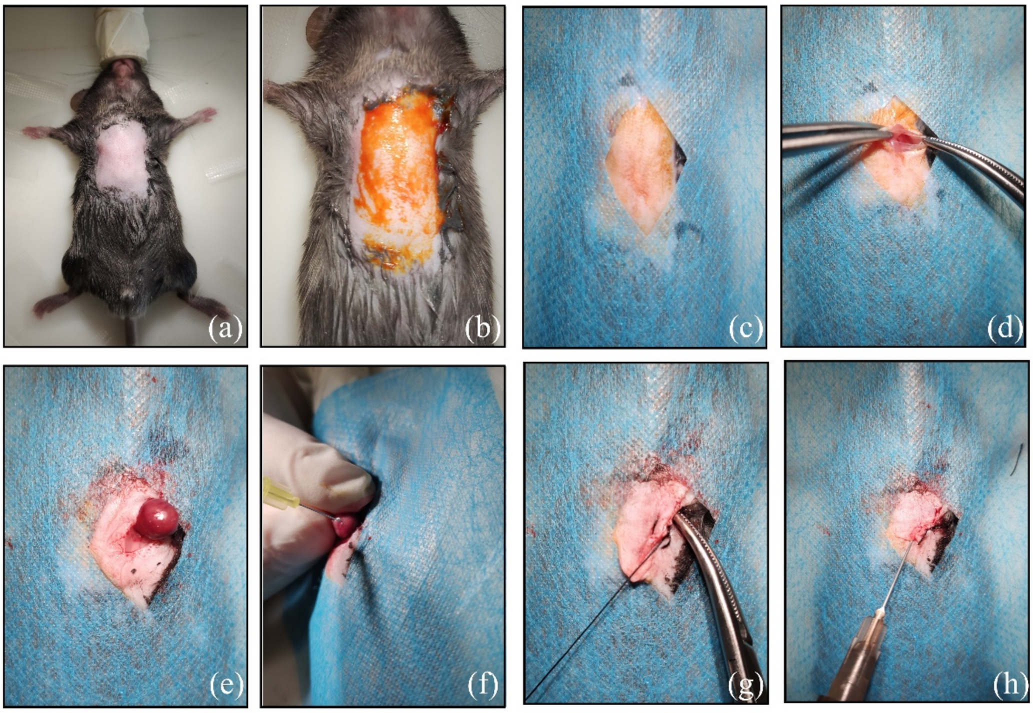

Supplementary Figure S1: Schema for intramyocardial injection of adeno-associated virus in mouse hearts. (a) The hair was removed by commercially available depilatory cream. (b) Sterilization of the operative site using several scrubs of povidone-iodine. (c) A sterile drape was employed to cover the operative site. (d) 0.5-cm skin incision along the line connecting axilla and xiphoid. (e) Hearts were exposed through left thoracotomy at the 5th intercostal space with the help of forceps and a micro-mosquito hemostat. (f) AAV was delivered via direct injection into the left ventricular wall via Hamilton syringe (5 sites, 10 μl/site). (g-h) Closure of the skin and analgesic treatment.

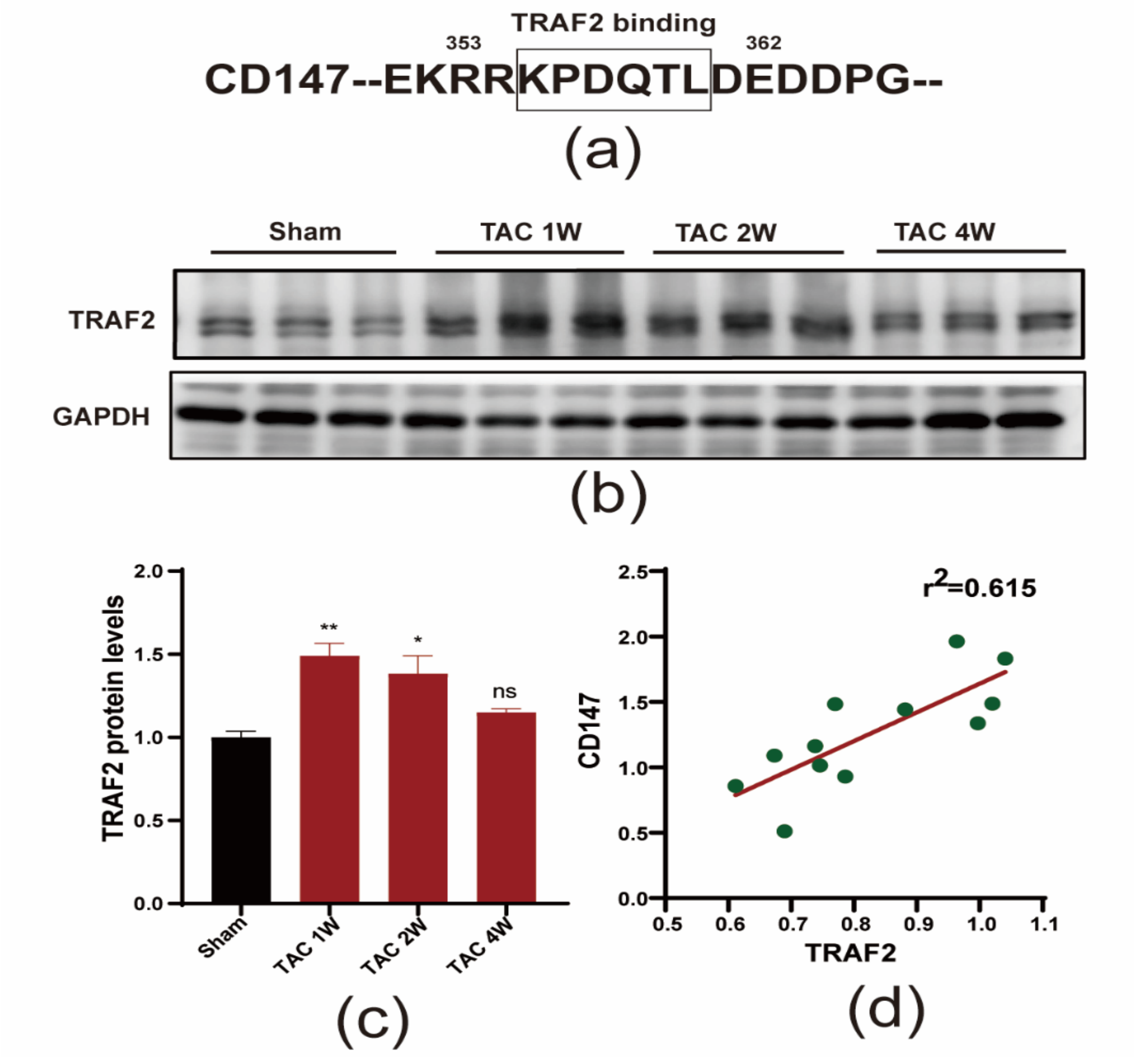

Supplementary Figure S2: TRAF2 was upregulated in mouse hearts after sustained pressure overload. (a) CD147 possesses TRAF2-binding motif in its cytoplasmic domain. (b-c) Representative immunoblotting and statistical analysis of TRAF2 expression levels in cardiac tissue after TAC in indicated time points (n=3). *P < 0.05 vs the Sham group; **P < 0.01 vs the Sham group. (d) Correlation analysis between CD147 and TRAF2 protein expression levels in response to pressure overload in indicated time points (n=3).

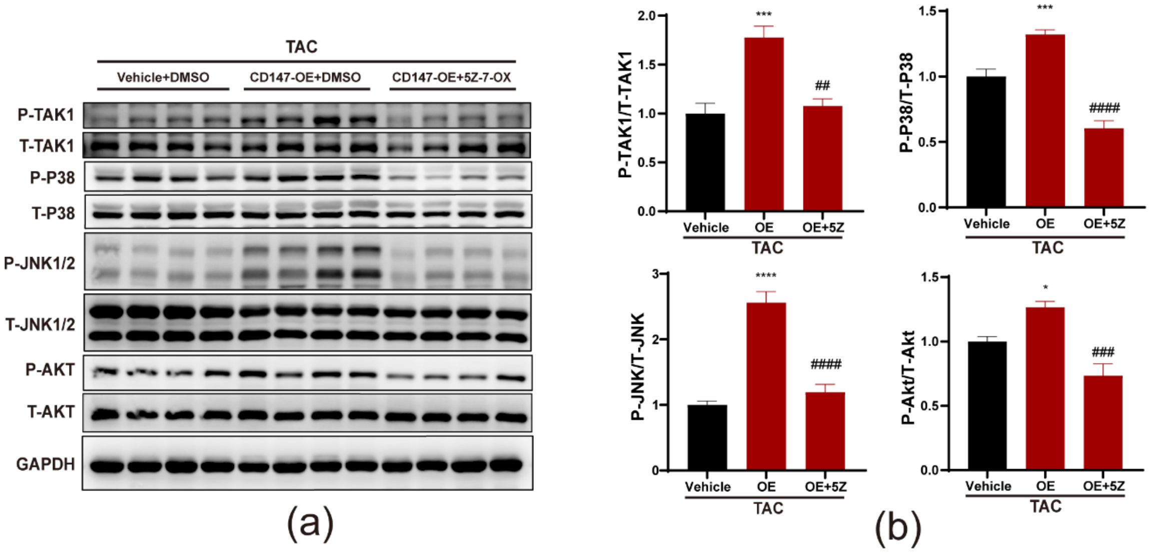

Supplementary Figure S3: Blocking TAK1 activity reduced the downstream activation of CD147-mediated TRAF2-TAK1 signalling. (a-b) Representative western blots and quantitative results of the phosphorylated and total protein levels of TAK1, P38, P-JNK1/2 and Akt in hearts from vehicle and CD147-OE mice after TAC treated DMSO or 5Z-7-ox (n=4). *P < 0.05 vs the Vehicle group; ***P < 0.001 vs the Vehicle group; ****P < 0.0001 vs the Vehicle group; #P < 0.05 vs the CD147-OE group; ##P < 0.01 vs the CD147-OE group; ###P < 0.001 vs the CD147-OE group; ####P < 0.0001 vs the CD147-OE group.

Supplementary Table S1. Mouse primers sequences for qRT-PCR

Primer	Sequences
ANP	Forward:5'- CGTGCCCCGACCCACGCCAGCATGG -3'<br>Reverse:5'- GCCTCCGAGGGCCAGCGAGCAGAGC -3'
BNP	Forward:5'- GAGGTCACTCCTATCCTCTGG -3'<br>Reverse:5'- GCCATTTCCTCCGACTTTTCTC -3'
α-MHC	Forward:5'- TGCACTACGGAAACATGAAGTT -3'<br>Reverse:5'- CGATGGAATAGTACACTTGCTGT -3'
β-MHC	Forward:5'- ACTGTCAACACTAAGAGGGTCA -3'<br>Reverse:5'- TTGGATGATTTGATCTTCCAGGG -3'
Collagen I	Forward:5'- GAGCGGAGAGTACTGGATCG -3'<br>Reverse:5'- TACTCGAACGGGAATCCATC -3'
Collagen III	Forward:5'- CCATAGCTGAACTGAAAACCACC -3'<br>Reverse:5'- CTGTAACATGGAAACTGGGGAAA -3'
MMP2	Forward:5'- CAAGTTCCCCGGCGATGTC -3'<br>Reverse:5'- TTCTGGTCAAGGTCACCTGTC -3'
CTGF	Forward:5'- GGGCCTCTTCTGCGATTTC -3'<br>Reverse:5'- ATCCAGGCAAGTGCATTGGTA -3'
GPX4	Forward:5'- GCCTGGATAAGTACAGGGGTT -3'<br>Reverse:5'- CATGCAGATCGACTAGCTGAG -3'
NOX4	Forward:5'- GAAGGGGTTAAACACCTCTGC -3'<br>Reverse:5'- ATGCTCTGCTTAAACACAATCCT -3'
FTH1	Forward:5'- CAAGTGCGCCAGAACTACCA -3'<br>Reverse:5'- GCCACATCATCTCGGTCAAAA -3'
GAPDH	Forward:5'- GAGGTCACTCCTATCCTCTGG -3'<br>Reverse:5'- GCCATTTCCTCCGACTTTTCTC -3'
